# Assessment of DDAH1 and DDAH2 Contributions to Psychiatric Disorders via In Silico Methods

**DOI:** 10.3390/ijms231911902

**Published:** 2022-10-07

**Authors:** Alena A. Kozlova, Anastasia N. Vaganova, Roman N. Rodionov, Raul R. Gainetdinov, Nadine Bernhardt

**Affiliations:** 1 Department of Psychiatry and Psychotherapy, University Hospital Carl Gustav Carus, Technische Universität Dresden, 01307 Dresden, Germany; 2 Institute of Translational Biomedicine, Saint-Petersburg State University, 199034 Saint-Petersburg, Russia; 3 Department of Internal Medicine III, Technische Universität Dresden, 01307 Dresden, Germany

**Keywords:** DDAH1, DDAH2, bipolar disorder, schizophrenia

## Abstract

The contribution of nitric oxide synthases (NOSs) to the pathophysiology of several neuropsychiatric disorders is recognized, but the role of their regulators, dimethylarginine dimethylaminohydrolases (DDAHs), is less understood. This study’s objective was to estimate DDAH1 and DDAH2 associations with biological processes implicated in major psychiatric disorders using publicly accessible expression databases. Since co-expressed genes are more likely to be involved in the same biologic processes, we investigated co-expression patterns with DDAH1 and DDAH2 in the dorsolateral prefrontal cortex in psychiatric patients and control subjects. There were no significant differences in DDAH1 and DDAH2 expression levels in schizophrenia or bipolar disorder patients compared to controls. Meanwhile, the data suggest that in patients, DDAH1 and DDHA2 undergo a functional shift mirrored in changes in co-expressed gene patterns. This disarrangement appears in the loss of expression level correlations between DDAH1 or DDAH2 and genes associated with psychiatric disorders and reduced functional similarity of DDAH1 or DDAH2 co-expressed genes in the patient groups. Our findings evidence the possible involvement of DDAH1 and DDAH2 in neuropsychiatric disorder development, but the underlying mechanisms need experimental validation.

## 1. Introduction

Mental disorders remain among the top ten leading causes of burden worldwide, which makes research to establish causal pathways imperative for effective prevention and treatment [1]. In recent years, nitric oxide (NO) signaling has been implicated in the pathophysiology of several mental illnesses, such as schizophrenia and affective disorders, comprising bipolar disorder and major depressive disorder [2]. For example, in schizophrenia patients, NO metabolism is impaired in various organs, including the brain [3,4,5], and high NO levels are found in post-mortem samples of the prefrontal cortex and hippocampus [6]. NO is a gaseous molecule acting as the second messenger of the NMDA receptor, thereby regulating glutamatergic transmission [7]. NO further interacts with the dopaminergic and serotonergic systems [8] and is involved in the storage, uptake, and release of transmitters, such as acetylcholine, noradrenaline, GABA, taurine, and glycine [9]. In the brain, NO regulates synaptic plasticity, neurodevelopment, and cerebral blood flow [10]. Meanwhile, excessive amounts of free radical NO lead to neurotoxicity and neurodegeneration [10,11].

There are three isoforms of enzymes generating NO: the neuronal (nNOS or NOS1), the inducible (iNOS or NOS2), and the endothelial NO synthases (eNOS or NOS3) [9]. Genetic variations in NOS genes have been associated with several psychiatric conditions: (i) NOS1 polymorphisms are associated with an increased risk of schizophrenia development [12]; (ii) NOS1 and NOS3 alleles are involved in modifying an individual’s susceptibility to bipolar disorder, depression, or risk of suicide attempts, and impact glutamatergic neurotransmission [13,14,15,16,17,18,19]; (iii) some NOS3 variants demonstrate a protective role in bipolar disorder [18]; (iv) NOS2 involvement in psychiatric diseases was demonstrated in animal models [1] and population studies [20], but the knowledge of this association is limited.

In addition, the NOS1 gene is methylated differently in schizophrenic patients and healthy individuals [21]. NOS1 coupling to the NMDA receptor is regulated by the NOS1 adapter protein (NOS1AP) [13], whose polymorphisms are associated with schizophrenia [22] and the severity of posttraumatic stress disorder [23]. Thus, there is increasing evidence that NOS1 and NOS3 are promising drug targets for treating schizophrenia and affective disorders [24,25,26].

NOS activity and NO levels are regulated by dimethylarginine dimethylaminohydrolases (DDAHs). There are two known isoforms whose amino acid sequences are 50% identical. DDAH1 is responsible for the degradation of N (omega), N (omega) dimethyl-L-arginine (ADMA), the major competitive inhibitor of NOS [27]. DDAH2 also contributes to the regulation of NO levels, although it is still being debated if through ADMA degradation or ADMA-independent mechanisms. Both isoforms of DDAHs are expressed in the brain in a regional and cell-type complementary fashion [28].

DDAH1 alleles are associated with the risk of developing autistic spectrum disorder or obsessive-compulsive disorder [29], and animal models of autism and schizophrenia endophenotypes present with increased DDAH1 levels [30,31]. At the same time, experiments in mice fed an Mg-restricted diet resulted in depression-like behavior and decreased DDAH1 expression [32]. However, the data on DDAH1 expression levels in different brain structures and psychiatric conditions are sparse. Thus far, reduced DDAH1 expression has been found in the anterior cingulate cortex in schizophrenic patients [33], whereas in the prefrontal cortex, a downregulation was transient and detectable only in the first years after the onset of the disease [34]. In addition, a strong downregulation of the hsa-miR-219-5p that is suggested to control DDAH1 expression was observed in schizophrenia patients [35].

DDAH2 gene variants are associated with schizophrenia and bipolar disorder susceptibility [36]. In schizophrenia patients, the DDAH2 gene is aberrantly methylated in both the prefrontal cortex and blood, and DDAH2 brain mRNA levels are significantly increased [37]. Further, loss of methylation was shown in schizophrenia patients with suicide attempts [38].

Loss or overexpression of NOS or other genes directly involved in this pathway may define altered NO-signaling in psychiatric disorders. The impact of the spatial expression pattern and the level of co-expressed genes may also be relevant. For example, malformation of NOS1 positive GABAergic interneurons was described in schizophrenia [39]. In addition, the mRNA for NPY, expressed by many NOS1/NADPH-d GABA-ergic neurons, is selectively decreased in neurons within the superficial white matter of subjects with psychosis. While NO facilitates blood flow through the cortical microvasculature, NPY mediates microvessel constriction; therefore, its deregulation leads to microcirculatory dysfunction [40].

Thus, associations of NOS gene mutations and expression deregulation with psychiatric disorders are well studied. However, relatively less is known about the contribution of other modulators of NO-mediated processes in the pathogenesis of these diseases. Suggesting that the context-dependent consequences of NO-signaling may differ in the cortex of psychiatric and non-psychiatric subjects, we attempted to estimate the differences of DDAHs co-expressed gene patterns in publicly available datasets. We performed a careful functional analysis of these co-expressed gene sets. Specifically, we evaluated evidence of DDAH1 and DDAH2 involvement in regulating processes associated with major psychotic disorders, schizophrenia, and bipolar disorder.

## 2. Results

### 2.1. DDAH1 and DDAH2 mRNAs Are Represented in the Dorsolateral Prefrontal Cortex in Non-Psychiatric Controls and Psychotic Patients

**Figure 1:**
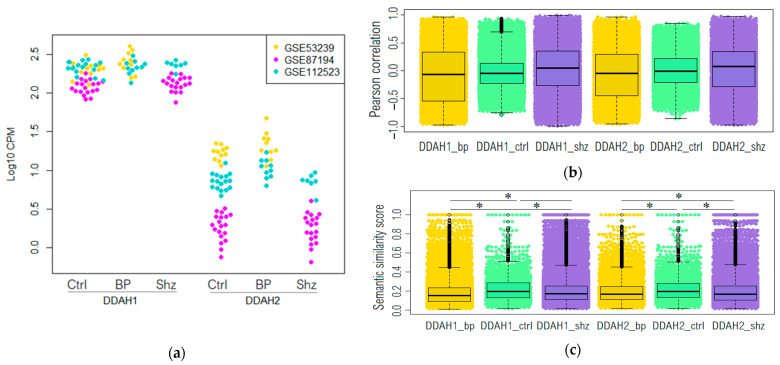
(**a**) Expression levels of DDAH1 and DDAH2 in the dorsolateral prefrontal cortex in non-psychiatric controls (Ctrl) and patients with bipolar affective disorder (BP) or schizophrenia (Shz) presented in three datasets: GSE53239 (yellow), GSE87194 (violet) and GSE112523 (blue). Beeswarm plot representing the distribution of correlation coefficients between DAAH1 or DAAH2 to all other genes expression levels in Brodmann area 46 in all groups (**b**) and the distribution of semantic similarity scores between the genes co-expressed (r > 0.8, *p* > 0.05) with DDAH1 or DDAH2 in all groups (**c**). * Games–Howell post hoc test *p* > 0.0001.

DDAH1 and DDAH2 mRNA were identified in all dorsolateral prefrontal cortex samples in the selected datasets (refer to Table 1) in both patients and non-psychiatric controls. The DDAH1 expression levels were greater than DDAH2. However, no significant differences in the DDAH1 and DDAH2 expression levels were identified when their expression was compared between the control group and patients with either bipolar affective disorder or schizophrenia (refer to Figure 1a). Slight upregulation of DDAH2 was identified in the tissue samples from patients with bipolar disorder compared to the control group in the GSE112523 dataset. However, this finding did not remain statistically significant after the adjustment (Padj > 0.05).

The DDAH1 and DDAH2 expression values are congruent in different datasets. DDAH2 expression is lower than DDAH1; however, its estimation is more prone to fluctuations and bias, particularly in the dataset GSE87194, where expression is lower than the other datasets.

The dataset GSE112523 combines data from subjects with schizophrenia, bipolar affective disorder, and non-psychiatric controls; thus, it was further analyzed to compare DDAH1 and DDAH2 co-expressed gene sets in these psychiatric disorders. The dataset contains dorsolateral prefrontal cortex samples (mainly BA46 area) of seven patients with schizophrenia, ten patients with bipolar disorders, and seventeen non-psychiatric control subjects. Study group data are summarized in Table 2.

### 2.2. Genome-Wide Co-Expression Analysis of DDAH1 and DDAH2 Co-Expressed Genes

The distribution of the Pearson correlation coefficient demonstrates that the median values for all three study groups fluctuate near the zero level. In addition, a predominance of positive correlation coefficients was observed for both DDAH1 and DAAH2 in the schizophrenia group (refer to Figure 1b).

### 2.3. Functional Analysis of DDAH1 and DDAH2 Co-Expressed Genes

Suggesting the semantic similarity score between two genes mirrors the functional linkage of these genes, the most DDAH1 and DAAH2 co-expressed genes (r > 0.8, *p* > 0.05) were selected for the comparative semantic similarity analysis of GO biological process terms, with which these genes were annotated. Genes selected based on the correlation levels were used as clusters for further analysis. Both DDAH1 and DDAH2 co-expressed genes in control subjects have higher (*p*  <  0.001) functional relationships compared to bipolar disorder or schizophrenic patients (refer to Figure 1c).

As a number of direct and indirect protein–protein interactions of DDAH1 and DDAH2 are identified and represented in public databases such as STRING [42], BioGRID [43], MINT [44], and HPRT [45,46], we compared our co-expression pattern with the data deposited in these resources. This approach also allows us to more precisely identify the genes, which may be functionally linked with DDAHs in our co-expressed gene sets and to select them for further analysis. We analyzed the protein–protein interaction databases to select all genes for which interactions with DDAH1 or DDAH2 were previously identified. From now on, we refer to these genes collectively as the “DDAH1 cluster” and “DDAH2 cluster”, respectively (see Material and Methods for the sources and cluster formation and Supplementary S1, S2 for the lists of genes included in these clusters).

**Figure 2:**
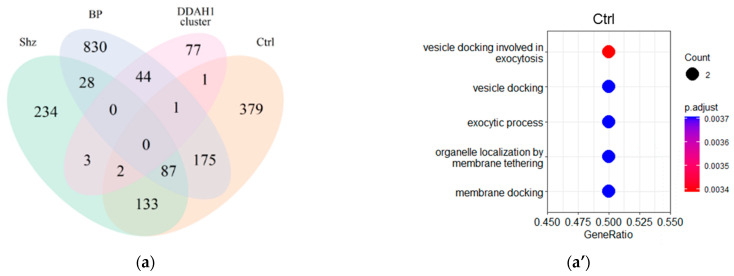

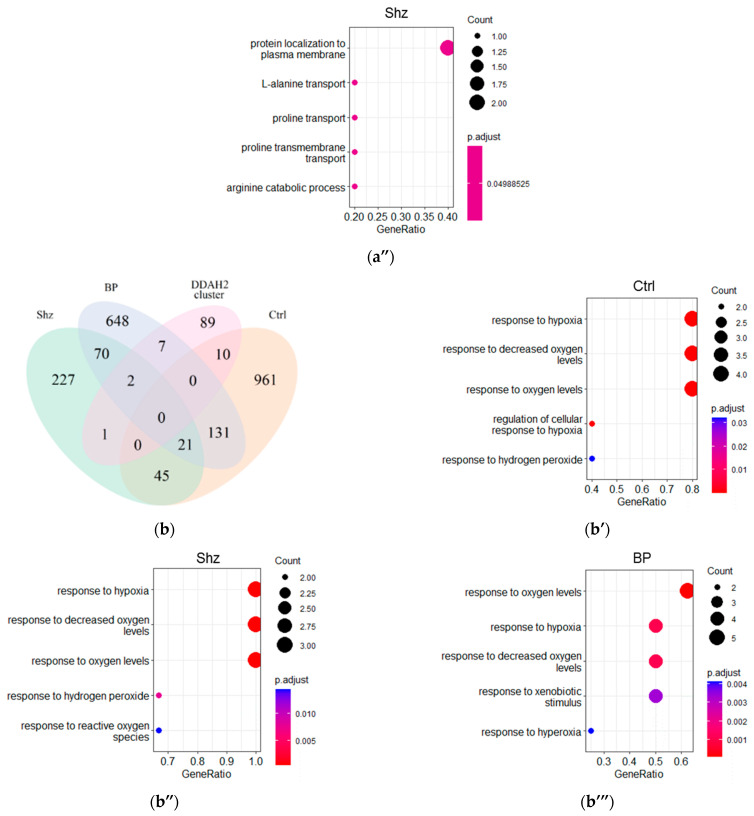
Expression of DDAH1 (**a**) and DDAH2 (**b**) interacting genes in the study groups. Venn diagram illustrating an overlay of genes co-expressed (r > 0.3, *p* > 0.05) with DDAH1 or DDAH2 in non-psychiatric controls (Ctrl), patients with bipolar disorder (BP), and schizophrenia (Shz) and the sets of genes involved in the functional interaction with DDAH1 and DDAH2 according to the public resources’ data. Gene ontology (GO) enrichment analysis of genes involved in the functional interaction with DDAH1 and DDAH2 according to the public resources’ data, which are co-expressed with DDAH1, top five of enriched GO groups are represented in controls (**a’**), patients with schizophrenia (**a”**), and DDAH2 in the controls (**b’**), patients with schizophrenia (**b”**), and patients with bipolar disorder (**b’”**). GO biological process terms showed no appropriate terms enrichment in the DDAH1 co-expressed gene cluster in patients with bipolar disorder.

To designate the genes of the “DDAH1 cluster” and “DDAH2 cluster” that are co-expressed with DDAH1 or DDAH2, respectively, in the control group, bipolar disorder patients and schizophrenia patients, we compared these clusters with the gene sets derived from our co-expression analysis (cut-off r > 0.3, *p* < 0.05). All study groups had low overlap between the “DDAH1 cluster” or “DDAH2 cluster” and sets of genes co-expressed with DDAH1 or DDAH2, respectively. However, Venn diagrams show that DDAH1 or DDAH2 co-expressed gene sets in each group include some genes involved in DDAHs-related biological processes. The greatest overlap between the co-expressed gene set and the “DDAH1 cluster” was identified in patients with bipolar affective disorder. Forty-five common genes were identified between these two gene sets (refer to Figure 2a). In contrast, the DDAH2 co-expressed gene set in controls includes ten genes from the “DDAH2 cluster”, whereas in the samples from patients with bipolar affective disorder and schizophrenia, the overlap was even lower (refer to Figure 2b).

### 2.4. GO Term Enrichment Results

Despite the small number of genes included in the “DDAH1 cluster” or “DDAH2 cluster” and co-expressed with DDAH1 or DDAH2, respectively, in the tissue samples studied in the analyzed dataset, we performed GO term enrichment analysis in these narrow gene subsets to explain their specific biological function. We found that the large group of genes for which involvement in DDAH1-related functions was previously identified (n = 45) is co-expressed with DDAH1 in the bipolar affective disorder group stochastically, and no significant GO term enrichment results were revealed in this gene set. In contrast, several GO groups were enriched in the constricted clusters of DDAH functionally associated genes, co-expressing with DDAH1 in schizophrenic patients and controls or with DDAH2 in all study groups.

In the group of schizophrenia patients, DDAH1 co-expressed genes of the “DDAH1-cluster” (n = 5) are found to associate with protein localization and amino-acid metabolism and transport (refer to Figure 2a”, Supplementary data S3, Figure S1A). In contrast, in the control group (n = 4), the terms describing exocytosis and cell response were predominant (refer to Figure 2a’, Supplementary data S3, Figure S1B). DDAH2 co-expressed and functionally linked gene cluster enrichment results were congruent in different groups, with specific features in all cases. The most enriched GO terms in the control group (n = 10) and patients (n = 9 in patients with bipolar disorder and n = 3 in schizophrenic patients) describe the response to hypoxic conditions (refer to Figure 2b’–b’”, Supplementary data S3, Figure S2A–C).

### 2.5. Identification of Enriched Transcription Factors and Other Protein Binding Motives in Promoters of DDAH1/DDAH2 Co-Expressed Genes

To uncover whether the DDAH1 and DDAH2 co-expressed genes are regulated by common transcription factors in patients and non-psychiatric controls, we compared the enriched transcription factors-binding motives in genes co-expressed with DDAH1 and DDAH2 (i.e., r > 0.3, *p* < 0.05), respectively, in the different study groups.

In healthy subjects, genes whose promoters contain short motif CG, which is recognized by zinc finger-CxxC proteins, are enriched in the DDAH1 co-expressed gene set (refer to Table 3, Supplementary data S4, Table S4.1). This association is completely lost in both the schizophrenia and bipolar disorder group. In the control groups’ transcriptomic data, genes whose promoters contain the AGGGGGA motif, which is recognized by several C2H2 zinc finger transcription factors, are enriched in the DDAH2 co-expressed gene set (refer to Table 3, Supplementary Sdata 4, Table S4.2). In contrast, in patients with bipolar affective disorder, several types of promoters, including the promoters C2H2 zinc finger transcription factors binding sites, are over-represented in the DDAH2 co-expressed gene set (refer to Table 3, Supplementary data S4, Table S4.3). In the meantime, we did not observe any over-represented motives in the promoters of DDAH2 co-expressed genes in patients with schizophrenia.

### 2.6. Disease Ontology Gene Set Enrichment Analysis

**Figure 3:**
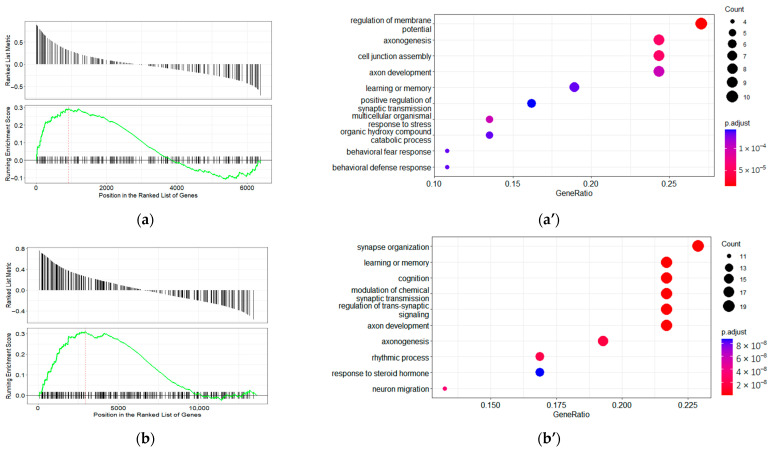

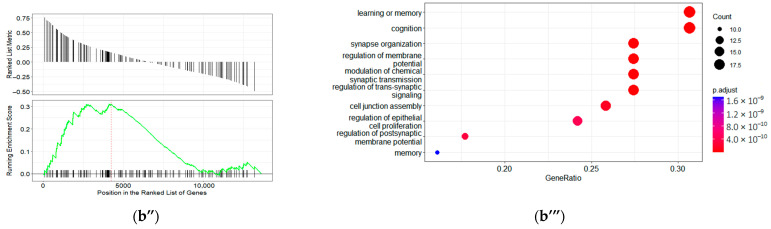
The genes involved in the developmental disorder of mental health co-expressed with DDAH1 and DDAH2 in non-psychiatric subjects. The enrichment plot for the representation of “DOID:0060037: developmental disorder of mental health” Disease Ontology (DO) term in the set of genes ranked based on their co-expression with DDAH1 in non-psychiatric subjects (**a**) and top 10 of biological processes in which enrichment core genes are implicated (**a’**). The enrichment plot for the representation of “DOID:0060037: developmental disorder of mental health” DO term (**b**) and “DOID:0060037: developmental disorder of mental health” DO term (**b”**). “DOID:0060041: autism spectrum disorder”/“DOID:12849: autistic disorder” in genes ranked based on their co-expression with DDAH2 in non-psychiatric subjects; (**b’**,**b’”**) top 10 of biological processes in which enrichment core genes for “DOID:0060037: developmental disorder of mental health” and “DOID:0060041:autism spectrum disorder”/“DOID:12849: autistic disorder” are implicated respectively.

For disease ontology terms, 74 terms that predominantly characterize gene involvement in neoplastic disease (benign tumors and cancer) were enriched in DDAH1 co-expressed genes in the control group (refer to Supplementary data S5, Table S5.1). In genes co-expressed with DDAH1 in samples from schizophrenia patients, five terms corresponding to non-cancerous disease were enriched (refer to Supplementary data S5, Table S5.2). No DO terms were enriched in genes co-expressed with DDAH1 in the patients with bipolar affective disorder. In the context of the mental health terms, only one term, “DOID:0060037: a developmental disorder of mental health” (refer to Figure 3a), is significantly over-represented in the set of genes co-expressed with DDAH1 in the control group. Genes that regulate membrane potential, axonogenesis, and cell junction assembly contribute most to the enrichment result for this term (i.e., enrichment core; refer to Figure 3a’). No other associations with mental disorders were identified in DDAH1 co-expressed genes in any study group.

Over one hundred DO terms were enriched in DDAH2 co-expressed gene groups in samples from patients with bipolar disorder and non-psychiatric subjects (refer to Supplementary data S5, Tables S5.3 and S5.4). Conversely, in genes co-expressed with DDAH2 in samples from schizophrenic patients, only eighteen terms are enriched (refer to Supplementary data S5, Table S5.5). As in the DDAH1 co-expressed gene set, genes of the term “DOID:0060037: a developmental disorder of mental health” are enriched in the control group. In addition, the genes corresponding to the term “DOID:0060041: autism spectrum disorder”/“DOID:12849:autistic disorder” are significantly over-represented in this set (refer to Figure 3b,b”). The functional characteristics of the enrichment core of “DOID:0060037: a developmental disorder of mental health” in DDAH2 co-expressed genes in non-psychiatric subjects differ slightly from DDAH1 co-expressed genes. The enrichment core genes are associated with synapse organization and cognitive functions such as learning, memory, and cognition (refer to Figure 3b’). Curiously, the top three over-represented functions in “DOID0060041:autism spectrum disorder”/“DOID:12849 autistic disorder” in the enrichment core are the same (refer to Figure 3b’”).

## 3. Discussion

This study found DDAH1 and DDAH2 expression in all dorsolateral prefrontal cortex samples from patients and non-psychiatric control subjects. The expression level of DDAH1 was considerably higher than the DDAH2 expression levels in all subjects. We did not observe changes in DDAH1 expression levels here. In line, normal DDAH1 expression levels have previously been shown in chronic schizophrenic patients, while DDAH1 upregulation was found in patients with short-term schizophrenia [33]. For DDAH2 expression, upregulation is reported in association with schizophrenia patient-specific methylations and promoter region SNPs [36]. Other studies have also shown that DDAH2 mRNA levels were significantly elevated in brain tissue in schizophrenia, although the brain region was not specified in this study [37]. DDAH2 expression upregulation has also been described in the prefrontal cortex of patients with bipolar disorder [47]. While a similar trend was found in the present study, it does not reach statistical significance. The discrepancy between our results and published data may be attributed to the differences in study groups and applied methods.

Affective disorders such as major depression or bipolar disorder are associated with an aberrant expression pattern of NOS in the dorsolateral prefrontal cortex. Deregulation does, however, not influence the expression level of NOS1-, NOS2-, or NOS3-mRNA in whole cortex samples, but it was accompanied by changes in protein localization in cortical layers [48]. Thus, NO deregulation in the brain of psychiatric patients does not solely depend on gene expression levels. It also may be associated with the disturbance of expression patterns in the complex multicellular cortex structure and disease-associated deregulation of biological processes in the cortex. Considering that the gene’s co-expression mirrors the similar biologic function of these genes [49], we attempted to compare DDAH1 and DDAH2 co-expression patterns in control subjects and patients with psychiatric disorders.

The Pearson correlation coefficient is useful for estimating gene co-expression [50], revealing that subsets of genes for which co-expression with DDAH1 or DDAH2 is predicted in different groups hardly overlap. As the GO semantic similarity score between genes is related to the involvement of their products in the protein–protein interaction network [51], the functional relationship between DDAH1 or DDAH2 co-expressed genes appears to be higher in non-psychiatric controls. This difference may be related to the deregulation of DDAH-associated processes. Further analysis of DDAH co-expressed genes may uphold this assumption.

To increase the stringency of the selection of DDAHs interacting partners in each study group, we selected genes with the correlation coefficient (r) > 0.3, *p* < 0.05, which are suggested to interact with DDAHs. The overlap between sets of genes that are co-expressed with DDAHs in different conditions and “DDAH1 cluster” or “DDAH2 cluster” was low. Meanwhile, the occurrence of low overlap between an experimentally identified gene co-expression pattern and protein–protein interaction data described in the literature or databases was observed and discussed in previous studies [52,53].

In the non-psychiatric control subjects, these genes are involved in vesicular transport in both directions. In contrast, in schizophrenic patients, this association is lost, and genes associated with protein localization and amino acid metabolism predominate. Enrichment of any biological process in DDAH1-interacting genes co-expressed with DDAH1 is completely lost in patients with bipolar disorder patients. The significance of NO-signaling for normal transcytosis functioning, i.e., vesicular traffic across the interior of cells in the blood–brain barrier, has been demonstrated [54]. In addition, NO diffusion stimulates the release of vesicles in the synaptic cleft [55]. Thus, the association of DDAH1 co-regulated genes with vesicle transport seems reasonable. Psychiatric disorders such as schizophrenia and bipolar disorder may be associated with brain-blood barrier dysfunction [56]. Disruption of eNOS is one of the suggested reasons for increased blood–brain barrier permeability [57]. Losing the association of DDAH1 co-expressed genes with the vesicular traffic (exocytosis and endosome trafficking) may mirror this or any other aspect of NO-dependent pathway disruption in the prefrontal cortex in psychiatric patients.

The association of DDAH2 co-expressed genes with the response to the hypoxic stress identified in this study is expected in light of considerable evidence of DDAH2 upregulation in hypoxic conditions. The growth of DDAH2 expression levels in response to hypoxia was described in monocytes [58,59], endothelium [60], and myotubes [61]. In the studied group, DDAH2 co-expressed genes in the prefrontal cortex are stably associated with the response to hypoxia, despite the psychiatric diagnosis.

Deregulation of DDAH1 and DDAH2-associated processes in psychiatric patients was also confirmed in the analysis of protein-binding motif enrichment in their promoter regions. In schizophrenic patients, the over-representation of any specific promoter motive is completely lost, both in DDAH1 and DDAH2 co-expressed genes. In patients with bipolar disorder, the enrichment is also lost in the DDAH1 co-expressed gene set. While, in genes co-expressed with DDAH2 in the prefrontal cortex of patients with bipolar disorder, several protein-binding patterns are significantly over-represented. Notably, AP-2 transcription factors play essential roles in sleep regulation in the nematode Caenorhabditis elegans and the fruit fly Drosophila melanogaster [62]. The AP-2 paralogous transcription factors Tfap2a and Tfap2b control sleep behavior in mice, allowing for bidirectional control of sleep quality [63]. Over-representation may thus link prefrontal DDAH2 functionality with sleep disturbance, a core symptom of bipolar disorder [64]. Genes co-expressed with DDAH1 in non-psychiatric subjects frequently harbor the CG motif in their promoters. Human CxxC-binding domains display different structures and selectivity [65]. The CG pattern is the sole DNA-binding domain of CGBP, which is implicated in the expression of genes associated with CpG islands and the regulation of cytosine methylation [66]. ShinyGO software also demonstrated the enrichment of TET1 CxxC-binding protein, which binds predominantly on CGCGAT motifs [65], whose role in the expression regulation is also dualistic. However, TET1 binds and represses CpG-rich promoters by interacting with the polycomb repressive complex 2 [67]. In the brain structure, it is involved in regulating synapse development and functioning, memory, neuronal death and repair, and neuro-glial communication. The lost association of DDAH1 expression with CG promoter motif genes may mirror the overexpression of TET1 in cortical structures in patients with schizophrenia or bipolar disorder [68]. As the co-expressed genes are suggested to share their function, the demonstrated loss of correlation between expression of DDAH1 and genes harboring CG patterns in their promoters may distort the DDAH1 role in memory, learning, and neuron functioning. Still, further experimental work is needed on this matter.

The C2H2 zinc finger MZF-1 binding pattern is over-represented in DDAH2 co-expressed genes in non-psychiatric subjects and patients with bipolar disorder, but in schizophrenic patients, this association is lost. The transcription factor MZF-1 is a known tumor suppressor [69]. A significant portion of genes specifically expressed in the cortex, hindbrain, and midbrain harbor MZF-1 binding sites in their promoters [70], but the significance of MZF-1 expression in the central nervous system remains not well understood. In oxygen–glucose deprivation conditions, MZF-1 mediates the protective effect of human umbilical cord blood cells on both neurons and oligodendrocytes in mixed cultures [71,72]. This transcription factor also seems involved in gene regulation after peripheral nerve injury [73]. However, in contrast to non-psychiatric controls and schizophrenia, many other enriched promoter motifs in genes co-expressed with DDAH2 are found in patients with bipolar disorder samples.

Of note, neither TET1 binds the DDAH1 promoter, nor does MZF1 regulate DDAH2 expression directly, as summarized in the Chip Seq Atlas [74] or Signaling Pathways Project [75]. Thus, the DDAHs co-expression with TET1- or MZF1-regulated genes needs an explanation, which is more complex than the co-regulation of the same transcription factors motivating further research in this direction.

Disease Ontology was designed for researchers to study gene–disease relationships [76]. It has a hierarchical structure [77]; thus, the “DOID:0060037: developmental disorder of mental health” term covers the term “DOID0060041: autism spectrum disorder” and other terms corresponding to a learning disability, intellectual disability, attention deficit hyperactivity disorder, communication disorder, eating disorder and some other specific developmental disorders. The co-expression of DDAH1 and DDAH2 with the genes annotated with these terms was identified in dorsolateral prefrontal cortex samples in non-psychiatric controls but was lost both in schizophrenic and bipolar disorder patients.

Developmental mental disorders, including autism spectrum disorders, demonstrate behavior and cognitive disabilities, which are also intrinsic to major psychoses [78]. At the same time, schizophrenia and bipolar disorder patients exhibit a developmental lag [79], and the deregulation of genes involved in neurogenesis and neurodifferentiation was identified in schizophrenia patients at disease onset [34]. The genetic and molecular backgrounds of these diseases share numerous similarities. Genes with a documented association with neurodevelopmental and neuropsychiatric disorders are predominantly involved in transcription, synaptic transmission, cell–cell communication, ion transmembrane transport, intracellular signaling pathways, cell cycle, metabolic processes, nervous system development, and neuron death [78,80,81,82,83]. The common genetic etiology mirrors the high comorbidity of these psychiatric diseases and developmental mental disorders [84]. Hence, in the latest version of the Diagnostic and Statistical Manual of Mental Disorders, Fifth Edition (DSM-5), schizophrenia has been listed in proximity to neurodevelopmental disorders [85]. Currently, schizophrenia and bipolar disorder may be considered neurodevelopmental disorders with a widening of the neurodevelopmental spectrum [86] the loss of co-expression between genes characterized by the term “DOID:0060037: a developmental disorder of mental health”, including genes involved in synaptogenesis, axonogenesis and cognitive functions, and DDAHs is of particular interest.

Thus far, most of the evidence on DDAH/ADMA axis relevance for neuropsychiatric disorders comes from case-control clinical studies and measurements of peripheral ADMA and NO levels without offering in-depth mechanistic insight. Nevertheless, connections between the DDAH/ADMA axis and oxidative stress markers, as well as molecules important for cognitive processes, have been shown and support the findings of this study. For example, plasma levels of the oxidative stress-induced lipid peroxidation product 4-HNE were increased and correlated positively with plasma ADMA levels in depression [87] and schizophrenia patients [88]. In terms of cognition, a correlation between plasma ADMA levels and cognitive deficits has been established, with decreases in ADMA levels leading to improvements in working memory and attention [89,90]. ADMA infusion decreases BDNF, a factor highly associated with cognitive functions [91]. Lastly, the G allele of DDAH2 (−449 G/C) was positively associated with leukoaraiosis and high ADMA levels [92]. Progression of leukoaraiosis, a condition frequently met in neuropsychiatric disorders, relates to cognitive decline and thus could explain the link of DDAH2-cluster with cognition (learning and memory, cognition, synapse organization). In addition, ADMA levels in patients with leukoaraiosis were significantly higher than those in healthy controls [93], and these high concentrations of ADMA were associated with cognitive dysfunction in leukoaraiosis patients [94].

Our findings must be seen with some limitations. (i) The transcriptomic datasets are generated with different sequencing depths. Although all measures were taken to normalize the data, full uniformity and overcoming batch effects are unattainable. (ii) Only a few datasets in the GEO [95] repository were relevant for the study. Further study groups are relatively small and heterogeneous, and patient information is restricted. (iii) The mRNA abundance has a limited capacity as the indicator of downstream expression. The gene expression level and activity of gene products depend on multiple factors, including RNA stability, modifications, the translation rate and protein turnover, localization in the cell, and availability of ligands or co-interacting proteins. (iv) In addition, the size of gene clusters selected for the GO enrichment analysis was small and sometimes appeared less than the clusters used for the enrichment analysis. However, considering that the enrichment results are more informative than raw data about the GO groups in which the identified genes are included, we include GO enrichment results in this paper. Despite this limitation, we received statistically significant results, with gene ratios of 0.5–1 for several GO groups. (v) The used protein–protein interactions databases STRING [42], BioGRID [43], and HPRT [45,46] may be misleading for DDAH2 co-expressed genes because they are based on text mining and the assumption that DDAH2 is purely metabolizing ADMA, which is still debated. (vi) The analyzed data were restricted to the dorsolateral prefrontal cortical area and therefore do not allow for generalization to other brain regions. (v) Finally, our study lacks experimental validation. Despite all these limitations, the present work provides preliminary evidence that DDAH1 and DDAH2 are co-regulated with genes involved in mental disorder development and derangement. Experimental studies are now needed for confirmation and to gain mechanistic insight.

## 4. Materials and Methods

### 4.1. Public Resources and Databases

The expression data were derived from the public database of Gene Expression Omnibus (GEO) [95]. We used the terms “schizophrenia” and “bipolar disorder”; the filter “Expression profiling by high throughput sequencing” for the series type; and the filter “Homo sapiens” for the organism. Datasets that comprise the prefrontal cortex samples were selected (refer to Table 1). Unfortunately, the data for other brain structures were unrepresented or represented by a single dataset, making it impossible to compare them, and we did not include them.

The dataset GSE112523 combining data for the subjects with schizophrenia, bipolar disorder, and non-psychiatric controls was selected for further analysis of DDAH1 and DDAH2 co-expressed genes. This dataset was generated by the 75 bp paired-end sequencing, which was performed on an Illumina NextSeq 500 sequencer [41].

The genes of DDAH1- and DDAH2-interacting proteins (“DDAH1 cluster” and “DDAH2 cluster”, refer to Supplementary data S1, S2) for the comparative analysis were selected from the public databases STRING [42], MINT [44], BioGRID [43], and HPRD [45,46]. STRING database was searched for the human data in the full STRING network (i.e., for both functional and physical protein associations), and filtered for the data received by the text mining, experiments, databases, co-expression, or co-occurrence by the basic settings options. BioGRID data were filtered for the interactions identified in human studies. All other databases were searched with default settings. Then, the genes of all identified proteins were included in the “DDAH1 cluster” or “DDAH2 cluster”.

### 4.2. Data Normalization and Statistical Analysis

Raw counts were count per million (CPM)-normalized by edgeR package [96]. CPM values above the threshold level 1 were considered positive. The distribution of CPM-normalized expression levels in the analyzed samples was visualized by the beeswarm R package.

To estimate the differential gene expression, raw counts were normalized using the Trimmed Mean of M-values (TMM) method by the edgeR package [96] to avoid batch effects. Differentially expressed genes were identified by the glmQLFTest test using the edgeR package [96]. *p* values were adjusted for multiple testing corrections using the Benjamini–Hochberg method. Genes were considered differentially expressed if adjusted *p* values (Padj) < 0.05.

### 4.3. Measurement of Co-Expression

Before co-expression measurement, CPM-normalized data were filtered to exclude genes that are expressed below the threshold (i.e., CPM = 1 for all samples in the study groups). Data for different study groups were filtered independently. DDAH1 and DDAH2 co-expressed genes were selected by Pearson’s correlation coefficient (r > 0.3, *p* < 0.05). Genes co-expressed with DDAH1 or DDAH2 in the different study groups were included in separate gene clusters (i.e., DAAH1-co-expressed genes in the control group, DAAH1-co-expressed genes in schizophrenic patients, DAAH1-co-expressed genes in patients with bipolar disorder, DAAH2-co-expressed genes in the control group, DAAH2-co-expressed genes in schizophrenic patients, and DAAH2-co-expressed genes in patients with bipolar disorder). The comparative analysis of the selected clusters was performed as described below.

### 4.4. Analysis of Functional Semantic Similarity between Genes

Gene Ontology (GO) semantic similarity was calculated by Wang’s method in the GOSemSim package [97] employing the “Biological process” GO terms. The difference in semantic similarity scores in different gene clusters was estimated by Brown–Forsythe and Games–Howell post hoc tests.

### 4.5. Function and Enrichment Analysis

The clusters of DDAHs co-expressed genes, identified in different study groups, were compared to each other, and to the “DDAH1 cluster” or “DAAH2 cluster”, which were identified by searching public databases as described above. DDAH1 co-expressed gene clusters were compared in the control group, schizophrenic patients, and patients with bipolar disorder, and then matched with the “DDAH1 cluster”. Similarly, the DDAH2 co-expressed gene clusters were evaluated. The overlap between identified gene clusters was visualized by the VennDiagram R package. To increase the stringency of DDAHs interacting patterns, the genes that were common for DDAHs co-expressed groups and the “DDAH1 cluster” or “DAAH2 cluster” were selected for further analysis, as it was described elsewhere [52,98].

Enriched motifs in promoters of the studied gene sets were identified using the ShinyGo 0.76 [99] web tool (available at http://bioinformatics.sdstate.edu/go/, accessed 10 June 2022). The upstream 300 bp region was specified as the promoter.

GO enrichment analysis (identification of GO terms that are significantly enriched by the genes of the selected set) was performed in the identified co-expressed gene clusters, and visualization of results was performed by the clusterProfiler Bioconductor package [100].

Disease Ontology (DO) enrichment analysis was performed in gene lists ranging from the highest value of the Pearson correlation of the gene expression level with the levels of DDAH1 or DDAH2 expression to the lowest. The clusterProfiler [100] and DOSE [101] R packages were used.

We considered significant enrichment results only for GO biological process terms, transcription factors, or DO terms with a false discovery rate value of <0.05.

## 5. Conclusions

Our results suggest a possible involvement of DDAH1 and DDAH2 in the pathophysiology of psychiatric disorders. While mRNA levels in the dorsolateral prefrontal cortex of psychiatric patients remain unchanged, a functional shift occurs that is reflected in dramatic changes in the expression of genes whose products interact with DDAH1/2. We found that correlations between expression levels of DDAH1 or DDAH2 and genes associated with mental illness are lost in cortical samples from psychiatric patients. DDAH1 and DDAH2 co-expressed genes were generally less integrated into shared functions in psychiatric patients than in non-psychiatric controls. Furthermore, the overlap between genes co-expressed with DDAHs in control subjects and psychiatric patients is low, possibly due to the complex deregulation of transcription factor activity. These data suggest that DDAHs are associated with the processes that form the molecular basis of mental and cognitive functions and thus may be potential therapeutic targets in psychiatric disorders.

## Figures and Tables

**Table 1:** Human transcriptomic GEO datasets meeting the inclusion criteria and included in the analysis.

Accession Number	Title	Diagnosis	Non-Psychiatric Controls (N)	Patients (N)
GSE53239	RNA-sequencing of the brain transcriptome implicates dysregulation of neuroplasticity, circadian rhythms, and GTPase binding in bipolar disorder	Bipolar affective disorder	11	10
GSE87194	Schizophrenia: post-mortem dorsolateral prefrontal cortex	Schizophrenia	19	19
GSE112523	DNA methylation in neurons from post-mortem brains in schizophrenia and bipolar disorder	Bipolar affective disorder	17	10
		Schizophrenia		7

**Table 2:** GSE112523 study groups’ demographic and clinical characteristics, data from [41].

Characteristics		Bipolar Disorder	Schizophrenia	Non-Psychiatric Controls
		n = 10	n = 7	n = 17
Gender	Male	7	6	12
	Female	3	1	5
Age	Median	47.7	45.1	45.8
	Range	29–77	29–55	31–68
Smoker status	Yes	8	2	5
	No	1	1	9
	Previous	0	0	1
	Unknown	1	4	2
Antipsychotic therapy	Yes	6	0	4
	No	4	17	3
Mood stabilizer therapy	Yes	6	0	0
	No	4	7	17

**Table 3:** Enriched promoter motifs in genes co-expressed with DDAH1 or DDAH2 in the dorsolateral prefrontal cortex of non-psychiatric control subjects, patients with bipolar disorder or schizophrenia.

Co-Expressed Genes	DDAH1		DDAH2	
	Number of Motives	Protein Families Which Bind the Enriched Motives	Number of Motives	Protein Families Which Bind the Enriched Motives
Non-psychiatric controls	1	CxxC	1	C2H2 ZF
Bipolar affective disorder	No enrichment		29	AP-2, bHLH, C2H2 ZF, CxxC, E2F, GCM, Nuclear receptor, Paired box
Schizophrenia	No enrichment		No enrichment	

## Data Availability

All whole tissue sample data are available in the GEO database (https://www.ncbi.nlm.nih.gov/geo/ (accessed on 10 December 2021); the detailed information is listed in Table 1).

## Supplementary Materials

The following supporting information can be downloaded at: https://www.mdpi.com/article/10.3390/ijms231911902/s1: S1. Title: DDAH1 cluster; S2. Title: DDAH2 cluster; S3. Title: Gene ontology (GO) enrichment analysis of genes involved in the functional interaction with DDAH1 or DDAH2: Figures S1 and S2; S4. Title: Enrichment analysis of promotor motifs in genes co-expressed with DDAH1 or DDAH2: Tables S4.1–S4.3; S5. Title: Disease Ontology (DO) terms enrichment analysis of genes co-expressed with DDAH1 or DDAH2: Tables S5.1–S5.5.
