# Supplementary material for: Assessment of DDAH1 and DDAH2 Contributions to Psychiatric Disorders via In Silico Methods
Source: Int J Mol Sci. 2022 Oct 7;23(19):11902. doi: 10.3390/ijms231911902 (PMC9569903; doi:10.3390/ijms231911902)
Supplement: Supplementary file 1 [file ijms-23-11902-s001.zip › ijms-1924321-supplementary (1).pdf]

International Journal of *Molecular Sciences*

MDPI

Supplementary Materials

Assessment of DDAH1 and DDAH2 Contribution in Psychiatric Disorders via In Silico Methods

Alena A. Kozlova [1,†], Anastasia N. Vaganova [2,†], Roman N. Rodionov [3], Raul R. Gainetdinov [2] and Nadine Bernhardt [1,*]

1 Department of Psychiatry and Psychotherapy, University Hospital Carl Gustav Carus, Technische Universität Dresden, 01307 Dresden, Germany
2 Institute of Translational Biomedicine, Saint-Petersburg State University, 199034 Saint-Petersburg, Russia
3 Department of Internal Medicine III, Technische Universität Dresden, 01307 Dresden, Germany
* Correspondence: Nadine.Bernhardt@uniklinikum-dresden.de
† These authors contributed equally to this work

S1. DDAH1 cluster

BioGRID

ADA, AFTPH, APEX1, APOOL, ARCN1, ATP5A1, BOLA3, CALM1, CATSPER4, CD69, CLIC1, CLIC4, CMPK1, CUL4A, DDAH2, ELAVL1, EPB41L2, FEN1, FGB, FSD1, GLOD4, HDHD3, HINT2, IQCB1, LACE1, LACTB2, LYRM4, MOV10, NDFIP2, NDUFAB1, NENF, NME1-NME2, NXF1, OBSL1, PARK7, PDP1, PGLS, POTEF, PRKAR1B, PSMD10, TMEM173, TRIM25, UCHL1, VCP,

STRING

ACE, AGXT2, ARG1, ARG2, B3GALT2, BRDT, BROX, C1orf52, COL11A1, CRACR2A, DDAH2, GSTK1, GSTZ1, LYPLA1, LYPLA2, NOS1, NOS3, PADI4, PRMT1, PRMT3, PRRC2A, PRRT1, RAB10, RAB12, RAB13, RAB15, RAB28, RAB40A, RAB40AL, RAB40B, RAB40C, RAB44, RAB8A, RAB8B, RASEF, RXRG, SKIV2L, SLC1A4, SLC1A5, SLC1A6, SLC1A7, VAV3, ZBTB12, ZNHIT6

MINT

YWHAH

S2. DDAH2 cluster

BioGRID

ACTN3, ANXA7, ATP1B1, BID, C21ORF33, CDC73, CDK4, CDKN1A, CLIC4, CLNS1A, DAOA, DDAH1, DDX58, DNAJB11, ENG, ENO1, EPB41L1, EPB41L2, EPB41L3, FANCL, FKBP1B, FN1, GADD45G, GOT1, GPATCH4, HCFC1, HDHD3, HINT1, HINT2, HNRNPH1, HYI, IGFN1, LACTB2, MKNK1, MPST, NDUFAB1, NFKBIA, NOXA1, NSP7AB, OGT, PARK7, PDP1, PIN1, PPA1, PRKAB2, PRNP, PRSS2, RAB11FIP5, RAP1B, RCC1, RPS6KA1, RPS6KA5, SARNP, SCGN, SLC25A35, SMN1, SOX2, STK3, TAF1D, TK1, TNFSF11, TRIM41, TRIP13, TSC22D1,
TULP2, VAC14, VCAM1, ZAK

STRING

ACE, AGPAT1, AGXT2, ARFGAP2, ARFGAP3, ARG1, ARG2, ATF6B, C6orf25, CAPN2, CAT, CDK2, CLIC1, CLNS1A, CRACR2A, CROCC, DDAH1, DOC2A, EPB41L1, EPB41L2, EPB41L3, EXOC7, GCHFR, HNRNPL, KIF5B, MLF2, MPO, NOS1, NOS3, PADI4, PAIP2, PBX2, PGLS, PRMT1, PRMT2, PRMT3, RBKS, RING1, SCGN, SLC1A7, SLC7A4, SNAP23, TOM1L1, TRIP13, UBL7, VEGFB, YRDC,

MINT

ANXA7, ATP1B1, CDC73, CDK4, CDKN1A, MAP3K20, TAF1D, MKNK1, SMN2, PIN1, STK3, TK1, GADD45G, RPS6KA5, DNAJB11, RAP1B, RCC1, RPS6KA1, TNFSF11, TSC22D1

HPRD

TRIP13

S3. Gene ontology (GO) enrichment analysis of genes involved in the functional interaction with DDAH1 or DDAH2

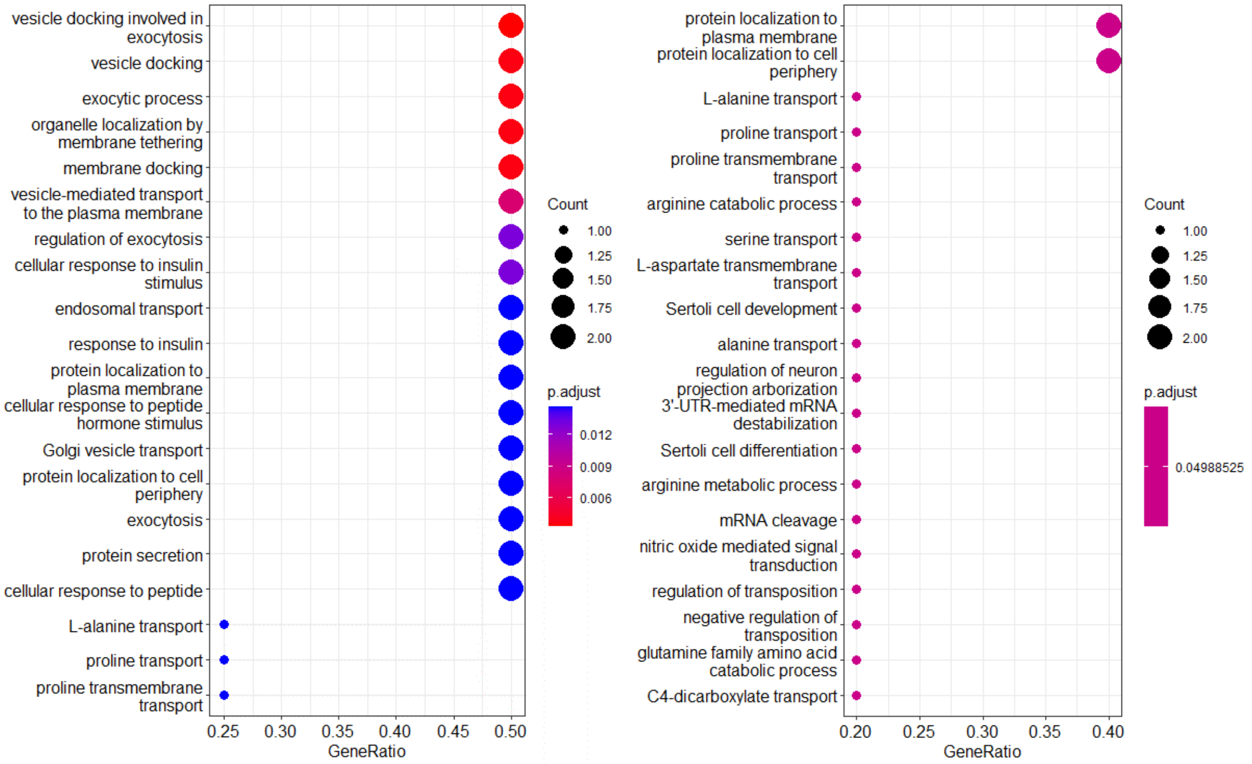

(**a**) (**b**)

Figure S1. GO enrichment analysis of genes involved in the functional interaction with DDAH1 according to the public resources' data which are co-expressed with (**a**) DDAH1 in control group and (**b**) DDAH1 in schizophrenic group.

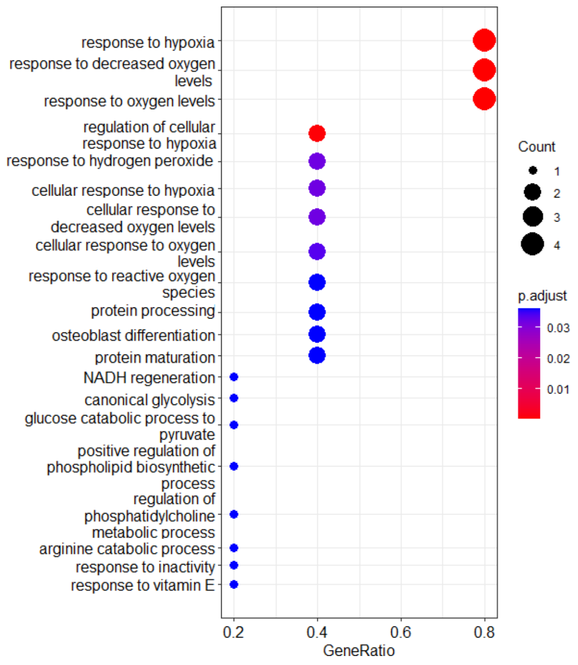

(**a**)

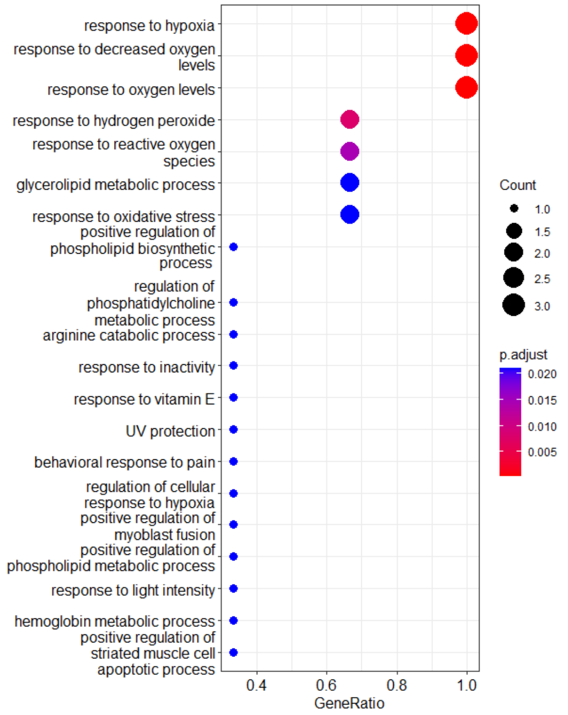

(**b**)

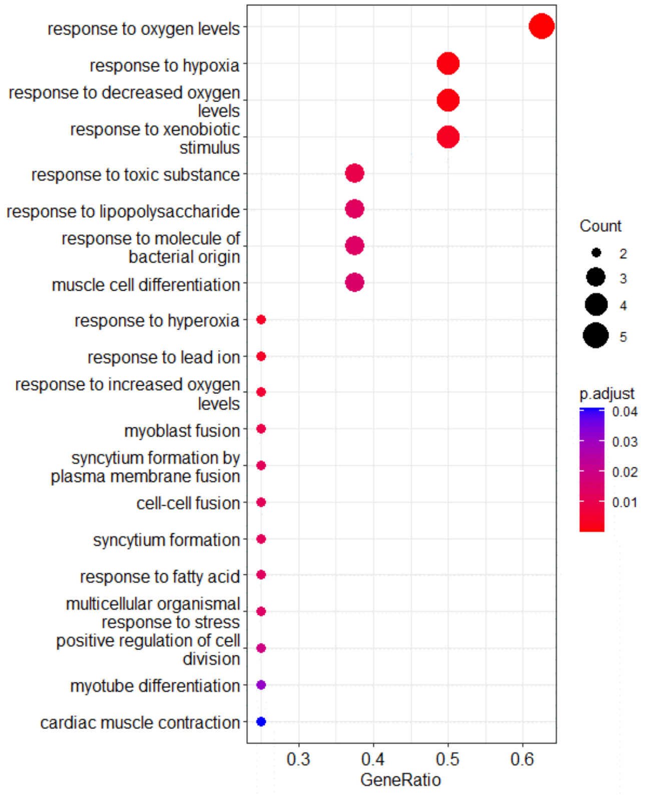

(**c**)

Figure S2. GO enrichment analysis of genes involved in the functional interaction with DDAH2 according to the public resources' data which are co-expressed with (**a**) DDAH2 in control group, (**b**) DDAH2 in schizophrenic group, (**c**) DDAH2 in bipolar affective disorder group.

S4. Enrichment analysis of promotor motifs in genes co-expressed with DDAH1 or DDAH2

Table S4.1. Enriched promoter motifs in genes co-expressed with DDAH1 in the dorsolateral pre- frontal cortex of non-psychiatric control subjects.

Enriched motif in promoter	TF	TF family	P val.	FDR	Score
CG	CGBP	CxxC	$2.4E^{-05}$	$1.0E^{-02}$	$5.0E^{+00}$
CG	TET1	CxxC	$7.5E^{-05}$	$1.5E^{-02}$	$5.0E^{+00}$

Table S4.2. Enriched promoter motifs in genes co-expressed with DDAH2 in the dorsolateral pre- frontal cortex of non-psychiatric control subjects.

Enriched motif in promoter	TF	TF family	P val.	FDR	Score
AGGGGGA	MZF1	C2H2 ZF	$1.7E^{-05}$	$6.9E^{-03}$	$3.2E^{+01}$

Table S4.3. Enriched promoter motifs in genes co-expressed with DDAH2 in the dorsolateral pre- frontal cortex of patients with bipolar disorder.

Enriched motif in promoter	TF	TF family	P val.	FDR	Score
GGGGGTGG	ZNF281	C2H2 ZF	$2.8E^{-09}$	9.0E-07	$2.3E^{+01}$
GGGGGGT	ZIC5	C2H2 ZF	$4.3E^{-09}$	9.0E-07	$1.6E^{+01}$
CCG	DNMT1	CxxC	$5.1E^{-08}$	7.1E-06	$1.1E^{+01}$
CCCCGGGC	TFAP2A	AP-2	1.2E-07	1.3E-05	1.4E+01
GCGGGGGCGGGG	EGR1	C2H2 ZF	3.6E-07	2.6E-05	3.4E+01
GGAGGAGGAGGGGGAGGAGG	ZNF263	C2H2 ZF	3.8E-07	2.6E-05	3.4E+01
GGGGGGGGGCC	PATZ1	C2H2 ZF	2.5E-06	1.5E-04	2.5E+01
GGGGGCGGGG	SP1	C2H2 ZF	4.0E-06	2.1E-04	2.5E+01
CCCCGGGC	TFAP2C	AP-2	7.0E-06	3.2E-04	1.5E+01
TGACCTCTGACCCC	NR2C2	Nuclear receptor	7.7E-06	3.2E-04	1.9E+01
GGGGCCCAAGGGGG	PLAG1	C2H2 ZF	$1.0E^{-05}$	3.8E-04	2.8E+01
TGCGGG	GCM1	GCM	$3.1E^{-05}$	1.1E-03	8.0E+00
GGGGGCGGGGC	SP2	C2H2 ZF	$3.6E^{-05}$	1.1E-03	2.7E+01
GGGG	ZNF202	C2H2 ZF	$5.4E^{-05}$	1.6E-03	7.0E+00
CG	CGBP	CxxC	1.2E-04	3.2E-03	5.0E+00
GGGCGGGGC	KLF5	C2H2 ZF	1.3E-04	3.3E-03	1.8E+01
CGAGGCGCAGTGATGCGTAGCGGC	PAX5	Paired box	1.9E-04	4.7E-03	1.6E+01
TGTCAGGGGGC	INSM1	C2H2 ZF	2.1E-04	4.8E-03	1.5E+01

GGCGCC	E2F2	E2F	2.3E-04	5.0E-03	1.2E+01
GCCTGAGG	TFAP2E	AP-2	2.8E-04	5.8E-03	1.1E+01
TCCCTGGGGA	EBF1	bHLH	3.0E-04	6.0E-03	1.2E+01
GCCTCAGGGCA	TFAP2A	AP-2	5.3E-04	9.9E-03	1.4E+01
GAGGGGGAA	MZF1	C2H2 ZF	7.2E-04	1.2E-02	1.2E+01
GGCGCTGTCCGTGGTGCTGAA	REST	C2H2 ZF	7.2E-04	1.2E-02	2.0E+01
GCCCTGGGGC	TFAP2C	AP-2	7.5E-04	1.2E-02	1.3E+01
GGGGA	MZF1	C2H2 ZF	8.4E-04	1.3E-02	9.0E+00
GGGAGT	EP300	Unknown	8.8E-04	1.4E-02	8.0E+00
ATGCGTGGGCGG	EGR4	C2H2 ZF	1.0E-03	1.5E-02	1.5E+01
GGGGCGCAGCTGCGCCC	NHLH1	bHLH	1.2E-03	1.7E-02	1.6E+01
GGGCGGGAA	E2F6	E2F	1.2E-03	1.7E-02	1.3E+01

S5. Disease ontology (DO) terms enrichment analysis of genes co-expressed with DDAH1 or DDAH2

Table S5.1. Enriched DO terms in genes co-expressed with DDAH1 in the dorsolateral prefrontal cortex of non-psychiatric control.

ID	Description	Enrichment score	*p*-value	p.adjust
DOID:114	heart disease	0,4183	<0,0001	0,0002
DOID:1287	cardiovascular system disease	0,3218	<0,0001	0,0008
DOID:0050155	sensory system disease	0,3756	<0,0001	0,0013
DOID:0050828	artery disease	0,3343	<0,0001	0,0014
DOID:684	hepatocellular carcinoma	0,3207	<0,0001	0,0014
DOID:686	liver carcinoma	0,3207	<0,0001	0,0014
DOID:3908	non-small cell lung carcinoma	0,3885	<0,0001	0,0014
DOID:3571	liver cancer	0,3183	0,0002	0,0017
DOID:305	carcinoma	0,3254	0,0002	0,0017
DOID:3905	lung carcinoma	0,3679	0,0002	0,0017
DOID:10763	hypertension	0,3699	0,0002	0,0017
DOID:3856	male reproductive organ cancer	0,3644	0,0002	0,0017
DOID:1749	squamous cell carcinoma	0,3371	0,0002	0,0017

DOID:2531	hematologic cancer	0,2957	0,0003	0,0020
DOID:193	reproductive organ cancer	0,3257	0,0003	0,0020
DOID:5614	eye disease	0,3669	0,0004	0,0020
DOID:0050013	carbohydrate metabolism disease	0,3458	0,0004	0,0020
DOID:4194	glucose metabolism disease	0,3458	0,0004	0,0020
DOID:17	musculoskeletal system disease	0,3029	0,0004	0,0020
DOID:557	kidney disease	0,3731	0,0004	0,0021
DOID:18	urinary system disease	0,3688	0,0005	0,0023
DOID:178	vascular disease	0,3080	0,0005	0,0024
DOID:1909	melanoma	0,3243	0,0006	0,0024
DOID:0060083	immune system cancer	0,2959	0,0006	0,0024
DOID:1492	eye and adnexa disease	0,3544	0,0007	0,0028
DOID:10283	prostate cancer	0,3572	0,0007	0,0028
DOID:3996	urinary system cancer	0,3430	0,0008	0,0028
DOID:0080001	bone disease	0,3252	0,0008	0,0028
DOID:0014667	disease of metabolism	0,3018	0,0010	0,0033
DOID:0050615	respiratory system cancer	0,3320	0,0014	0,0045
DOID:1240	leukemia	0,2993	0,0014	0,0045
DOID:0060158	acquired metabolic disease	0,3099	0,0015	0,0047
DOID:2994	germ cell cancer	0,3591	0,0016	0,0047
DOID:65	connective tissue disease	0,3012	0,0017	0,0049
DOID:1324	lung cancer	0,3296	0,0021	0,0060
DOID:170	endocrine gland cancer	0,3275	0,0024	0,0065
DOID:4451	renal carcinoma	0,3434	0,0026	0,0068
DOID:1037	lymphoblastic leukemia	0,3440	0,0027	0,0070
DOID:0060100	musculoskeletal system cancer	0,3375	0,0033	0,0079
DOID:9351	diabetes mellitus	0,3257	0,0033	0,0079
DOID:263	kidney cancer	0,3364	0,0033	0,0079

DOID:0060072	benign neoplasm	0,3220	0,0036	0,0084
DOID:10155	intestinal cancer	0,3615	0,0047	0,0106
DOID:4450	renal cell carcinoma	0,3511	0,0048	0,0106
DOID:3342	bone inflammation disease	0,3116	0,0051	0,0110
DOID:848	arthritis	0,3139	0,0069	0,0143
DOID:5672	large intestine cancer	0,3549	0,0070	0,0143
DOID:9256	colorectal cancer	0,3549	0,0070	0,0143
DOID:2914	immune system disease	0,2833	0,0073	0,0146
DOID:219	colon cancer	0,3491	0,0080	0,0156
DOID:1793	pancreatic cancer	0,3482	0,0084	0,0160
DOID:201	connective tissue cancer	0,3211	0,0085	0,0160
DOID:374	nutrition disease	0,3474	0,0106	0,0192
DOID:657	adenoma	0,3404	0,0106	0,0192
DOID:688	embryonal cancer	0,3305	0,0111	0,0198
DOID:0080000	muscular disease	0,3208	0,0122	0,0213
DOID:0060056	hypersensitivity reaction disease	0,3022	0,0131	0,0225
DOID:423	myopathy	0,3184	0,0143	0,0235
DOID:66	muscle tissue disease	0,3184	0,0143	0,0235
DOID:0060084	cell type benign neoplasm	0,3137	0,0144	0,0235
DOID:3118	hepatobiliary disease	0,3078	0,0152	0,0243
DOID:3093	nervous system cancer	0,2898	0,0158	0,0248
DOID:1612	breast cancer	0,2966	0,0162	0,0248
DOID:5093	thoracic cancer	0,2966	0,0162	0,0248
DOID:5679	retinal disease	0,3368	0,0170	0,0257
DOID:3459	breast carcinoma	0,3163	0,0191	0,0284
DOID:0050739	autosomal genetic disease	0,2842	0,0197	0,0287
DOID:74	hematopoietic system disease	0,3171	0,0199	0,0287
DOID:1579	respiratory system disease	0,3167	0,0217	0,0308

DOID:850	lung disease	0,3147	0,0230	0,0322
DOID:0060037	developmental disorder of mental health	0,2930	0,0235	0,0325
DOID:120	female reproductive organ cancer	0,3029	0,0290	0,0395
DOID:409	liver disease	0,2995	0,0309	0,0415
DOID:4766	embryoma	0,3187	0,0345	0,0457

Table S5.2. Enriched DO terms in genes co-expressed with DDAH1 in the dorsolateral prefrontal cortex of schizophrenic patients.

ID	Description	Enrichment score	*p*-value	p.adjust
DOID:0050155	sensory system disease	0.2820	0.0004	0.025
DOID:848	arthritis	0.27	0.0006	0.025
DOID:114	heart disease	0.2813	0.0007	0.025
DOID:3342	bone inflammation disease	0.2603	0.0008	0.025
DOID:10763	hypertension	0.2752	0.0012	0.0293

Table S5.3. Enriched DO terms in genes co-expressed with DDAH2 in the dorsolateral prefrontal cortex of non-psychiatric control subjects.

ID	Description	Enrichment score	*p*-value	p.adjust
DOID:0050013	carbohydrate metabolism disease	0,4882	< 0.0001	< 0.0001
DOID:0050155	sensory system disease	0,4803	< 0.0001	< 0.0001
DOID:0050161	lower respiratory tract disease	0,5993	< 0.0001	< 0.0001
DOID:0050177	monogenic disease	0,4033	< 0.0001	< 0.0001
DOID:0050615	respiratory system cancer	0,4825	< 0.0001	< 0.0001
DOID:0050737	autosomal recessive disease	0,4767	< 0.0001	< 0.0001
DOID:0050739	autosomal genetic disease	0,3999	< 0.0001	< 0.0001
DOID:0060056	hypersensitivity reaction disease	0,5306	< 0.0001	< 0.0001
DOID:0060072	benign neoplasm	0,4666	< 0.0001	< 0.0001
DOID:0060084	cell type benign neoplasm	0,4626	< 0.0001	< 0.0001
DOID:0060100	musculoskeletal system cancer	0,5322	< 0.0001	< 0.0001
DOID:0070004	myeloma	0,5692	< 0.0001	< 0.0001
DOID:0080000	muscular disease	0,4405	< 0.0001	< 0.0001

DOID:10155	intestinal cancer	0,4995	< 0.0001	< 0.0001
DOID:10283	prostate cancer	0,4838	< 0.0001	< 0.0001
DOID:1037	lymphoblastic leukemia	0,4687	< 0.0001	< 0.0001
DOID:104	bacterial infectious disease	0,6172	< 0.0001	< 0.0001
DOID:1040	chronic lymphocytic leukemia	0,5569	< 0.0001	< 0.0001
DOID:10534	stomach cancer	0,5276	< 0.0001	< 0.0001
DOID:10591	pre-eclampsia	0,5506	< 0.0001	< 0.0001
DOID:10763	hypertension	0,4986	< 0.0001	< 0.0001
DOID:1115	sarcoma	0,5668	< 0.0001	< 0.0001
DOID:114	heart disease	0,5011	< 0.0001	< 0.0001
DOID:1192	peripheral nervous system neoplasm	0,4383	< 0.0001	< 0.0001
DOID:120	female reproductive organ cancer	0,5136	< 0.0001	< 0.0001
DOID:1324	lung cancer	0,4882	< 0.0001	< 0.0001
DOID:1492	eye and adnexa disease	0,4754	< 0.0001	< 0.0001
DOID:15	reproductive system disease	0,5538	< 0.0001	< 0.0001
DOID:1579	respiratory system disease	0,5926	< 0.0001	< 0.0001
DOID:16	integumentary system disease	0,5534	< 0.0001	< 0.0001
DOID:1612	breast cancer	0,4884	< 0.0001	< 0.0001
DOID:170	endocrine gland cancer	0,4853	< 0.0001	< 0.0001
DOID:1749	squamous cell carcinoma	0,5138	< 0.0001	< 0.0001
DOID:1781	thyroid cancer	0,4945	< 0.0001	< 0.0001
DOID:1793	pancreatic cancer	0,4968	< 0.0001	< 0.0001
DOID:18	urinary system disease	0,5790	< 0.0001	< 0.0001
DOID:184	bone cancer	0,5436	< 0.0001	< 0.0001
DOID:1883	hepatitis C	0,5243	< 0.0001	< 0.0001
DOID:1909	melanoma	0,4770	< 0.0001	< 0.0001
DOID:193	reproductive organ cancer	0,4785	< 0.0001	< 0.0001
DOID:1936	atherosclerosis	0,6308	< 0.0001	< 0.0001

DOID:201	connective tissue cancer	0,5436	< 0.0001	< 0.0001
DOID:2043	hepatitis B	0,5957	< 0.0001	< 0.0001
DOID:2151	malignant ovarian surface	0,5384	< 0.0001	< 0.0001
DOID:2152	ovary epithelial cancer	0,5384	< 0.0001	< 0.0001
DOID:219	colon cancer	0,5029	< 0.0001	< 0.0001
DOID:2237	hepatitis	0,5233	< 0.0001	< 0.0001
DOID:2320	obstructive lung disease	0,6380	< 0.0001	< 0.0001
DOID:2348	arteriosclerotic cardiovascular disease	0,6327	< 0.0001	< 0.0001
DOID:2349	arteriosclerosis	0,6269	< 0.0001	< 0.0001
DOID:2355	anemia	0,5573	< 0.0001	< 0.0001
DOID:2394	ovarian cancer	0,5250	< 0.0001	< 0.0001
DOID:263	kidney cancer	0,5206	< 0.0001	< 0.0001
DOID:28	endocrine system disease	0,5002	< 0.0001	< 0.0001
DOID:2994	germ cell cancer	0,4784	< 0.0001	< 0.0001
DOID:3070	malignant glioma	0,5149	< 0.0001	< 0.0001
DOID:3083	chronic obstructive pulmonary disease	0,6477	< 0.0001	< 0.0001
DOID:3093	nervous system cancer	0,4255	< 0.0001	< 0.0001
DOID:3118	hepatobiliary disease	0,5262	< 0.0001	< 0.0001
DOID:3342	bone inflammation disease	0,5541	< 0.0001	< 0.0001
DOID:3347	osteosarcoma	0,5267	< 0.0001	< 0.0001
DOID:3393	coronary artery disease	0,5434	< 0.0001	< 0.0001
DOID:3459	breast carcinoma	0,5127	< 0.0001	< 0.0001
DOID:37	skin disease	0,5787	< 0.0001	< 0.0001
DOID:374	nutrition disease	0,4935	< 0.0001	< 0.0001
DOID:3856	male reproductive organ cancer	0,4767	< 0.0001	< 0.0001
DOID:3905	lung carcinoma	0,5060	< 0.0001	< 0.0001
DOID:3908	non-small cell lung carcinoma	0,5061	< 0.0001	< 0.0001
DOID:3996	urinary system cancer	0,5172	< 0.0001	< 0.0001

DOID:4001	ovarian carcinoma	0,5384	< 0.0001	< 0.0001
DOID:409	liver disease	0,5163	< 0.0001	< 0.0001
DOID:417	hypersensitivity reaction type II disease	0,5370	< 0.0001	< 0.0001
DOID:4194	glucose metabolism disease	0,4882	< 0.0001	< 0.0001
DOID:423	myopathy	0,4338	< 0.0001	< 0.0001
DOID:4450	renal cell carcinoma	0,5156	< 0.0001	< 0.0001
DOID:4451	renal carcinoma	0,5284	< 0.0001	< 0.0001
DOID:4766	embryoma	0,4867	< 0.0001	< 0.0001
DOID:4905	pancreatic carcinoma	0,4984	< 0.0001	< 0.0001
DOID:4960	bone marrow cancer	0,5642	< 0.0001	< 0.0001
DOID:5093	thoracic cancer	0,4884	< 0.0001	< 0.0001
DOID:557	kidney disease	0,5822	< 0.0001	< 0.0001
DOID:5614	eye disease	0,4726	< 0.0001	< 0.0001
DOID:5672	large intestine cancer	0,5068	< 0.0001	< 0.0001
DOID:5679	retinal disease	0,4495	< 0.0001	< 0.0001
DOID:5844	myocardial infarction	0,5560	< 0.0001	< 0.0001
DOID:654	overnutrition	0,4977	< 0.0001	< 0.0001
DOID:66	muscle tissue disease	0,4338	< 0.0001	< 0.0001
DOID:688	embryonal cancer	0,4775	< 0.0001	< 0.0001
DOID:7148	rheumatoid arthritis	0,5733	< 0.0001	< 0.0001
DOID:74	hematopoietic system disease	0,5511	< 0.0001	< 0.0001
DOID:848	arthritis	0,5561	< 0.0001	< 0.0001
DOID:850	lung disease	0,6045	< 0.0001	< 0.0001
DOID:854	collagen disease	0,5533	< 0.0001	< 0.0001
DOID:9256	colorectal cancer	0,5072	< 0.0001	< 0.0001
DOID:934	viral infectious disease	0,4839	< 0.0001	< 0.0001
DOID:9351	diabetes mellitus	0,4851	< 0.0001	< 0.0001
DOID:9538	multiple myeloma	0,5662	< 0.0001	< 0.0001

DOID:9970	obesity	0,4912	< 0.0001	< 0.0001
DOID:2621	autonomic nervous system neoplasm	0,4267	< 0.0001	< 0.0001
DOID:769	neuroblastoma	0,4267	< 0.0001	< 0.0001
DOID:0060085	organ system benign neoplasm	0,4870	< 0.0001	< 0.0001
DOID:3963	thyroid carcinoma	0,4921	< 0.0001	< 0.0001
DOID:1036	chronic leukemia	0,5168	< 0.0001	< 0.0001
DOID:326	ischemia	0,4913	< 0.0001	< 0.0001
DOID:11934	head and neck cancer	0,4737	< 0.0001	< 0.0001
DOID:657	adenoma	0,4537	< 0.0001	< 0.0001
DOID:1542	head and neck carcinoma	0,4656	< 0.0001	< 0.0001
DOID:655	inherited metabolic disorder	0,4288	< 0.0001	< 0.0001
DOID:6000	congestive heart failure	0,4998	< 0.0001	< 0.0001
DOID:8466	retinal degeneration	0,4556	< 0.0001	< 0.0001
DOID:0050736	autosomal dominant disease	0,3740	< 0.0001	< 0.0001
DOID:10652	Alzheimer's disease	0,3564	< 0.0001	< 0.0001
DOID:680	tauopathy	0,3525	< 0.0001	< 0.0001
DOID:870	neuropathy	0,4166	< 0.0001	< 0.0001
DOID:231	motor neuron disease	0,3724	0,0002	0,0002
DOID:0050890	synucleinopathy	0,3642	0,0003	0,0003
DOID:14330	Parkinson's disease	0,3864	0,0004	0,0004
DOID:936	brain disease	0,3021	0,0006	0,0006
DOID:332	amyotrophic lateral sclerosis	0,3729	0,0009	0,0009
DOID:0060037	developmental disorder of mental health	0,3106	0,0009	0,0010
DOID:0060040	pervasive developmental disorder	0,3404	0,0015	0,0016
DOID:5683	hereditary breast ovarian cancer	0,3413	0,0022	0,0023
DOID:0060041	autism spectrum disorder	0,3112	0,0146	0,0152
DOID:12849	autistic disorder	0,3112	0,0146	0,0152

Table S5. 4. Enriched DO terms in genes co-expressed with DDAH1 in the dorsolateral prefrontal cortex of bipolar disorder patients.

ID	Description	Enrichment score	*p*-value	p.adjust
DOID:0050013	carbohydrate metabolism disease	0,3303	< 0,0001	< 0,0001
DOID:0050161	lower respiratory tract disease	0,3340	< 0,0001	< 0,0001
DOID:0050615	respiratory system cancer	0,2903	< 0,0001	< 0,0001
DOID:0060056	hypersensitivity reaction disease	0,3165	< 0,0001	< 0,0001
DOID:10763	hypertension	0,3069	< 0,0001	< 0,0001
DOID:114	heart disease	0,3231	< 0,0001	< 0,0001
DOID:1579	respiratory system disease	0,3452	< 0,0001	< 0,0001
DOID:16	integumentary system disease	0,3606	< 0,0001	< 0,0001
DOID:18	urinary system disease	0,3380	< 0,0001	< 0,0001
DOID:1936	atherosclerosis	0,3754	< 0,0001	< 0,0001
DOID:2237	hepatitis	0,3267	< 0,0001	< 0,0001
DOID:2348	arteriosclerotic cardiovascular disease	0,3754	< 0,0001	< 0,0001
DOID:2349	arteriosclerosis	0,3859	< 0,0001	< 0,0001
DOID:263	kidney cancer	0,3097	< 0,0001	< 0,0001
DOID:3118	hepatobiliary disease	0,3375	< 0,0001	< 0,0001
DOID:3342	bone inflammation disease	0,3858	< 0,0001	< 0,0001
DOID:3393	coronary artery disease	0,4062	< 0,0001	< 0,0001
DOID:3905	lung carcinoma	0,3225	< 0,0001	< 0,0001
DOID:3908	non-small cell lung carcinoma	0,3334	< 0,0001	< 0,0001
DOID:3996	urinary system cancer	0,3143	< 0,0001	< 0,0001
DOID:409	liver disease	0,3328	< 0,0001	< 0,0001
DOID:417	hypersensitivity reaction type II disease	0,3164	< 0,0001	< 0,0001
DOID:4194	glucose metabolism disease	0,3303	< 0,0001	< 0,0001
DOID:557	kidney disease	0,3437	< 0,0001	< 0,0001
DOID:655	inherited metabolic disorder	0,3719	< 0,0001	< 0,0001
DOID:7148	rheumatoid arthritis	0,3660	< 0,0001	< 0,0001

DOID:74	hematopoietic system disease	0,3379	< 0,0001	< 0,0001
DOID:848	arthritis	0,3790	< 0,0001	< 0,0001
DOID:850	lung disease	0,3353	< 0,0001	< 0,0001
DOID:9351	diabetes mellitus	0,3239	< 0,0001	< 0,0001
DOID:1324	lung cancer	0,2931	< 0,0001	< 0,0001
DOID:5844	myocardial infarction	0,3916	< 0,0001	< 0,0001
DOID:4450	renal cell carcinoma	0,3342	< 0,0001	< 0,0001
DOID:37	skin disease	0,3718	< 0,0001	< 0,0001
DOID:28	endocrine system disease	0,3421	< 0,0001	< 0,0001
DOID:0070004	myeloma	0,3478	< 0,0001	< 0,0001
DOID:104	bacterial infectious disease	0,4058	< 0,0001	< 0,0001
DOID:934	viral infectious disease	0,2651	< 0,0001	< 0,0001
DOID:4960	bone marrow cancer	0,3468	< 0,0001	< 0,0001
DOID:4451	renal carcinoma	0,3134	< 0,0001	< 0,0001
DOID:0050338	primary bacterial infectious disease	0,4176	< 0,0001	< 0,0001
DOID:5614	eye disease	0,2886	< 0,0001	< 0,0001
DOID:0050155	sensory system disease	0,2821	< 0,0001	< 0,0001
DOID:0060072	benign neoplasm	0,2704	< 0,0001	< 0,0001
DOID:1115	sarcoma	0,3747	< 0,0001	< 0,0001
DOID:2994	germ cell cancer	0,2957	< 0,0001	< 0,0001
DOID:1520	colon carcinoma	0,3965	< 0,0001	< 0,0001
DOID:1037	lymphoblastic leukemia	0,2980	< 0,0001	< 0,0001
DOID:170	endocrine gland cancer	0,2715	< 0,0001	< 0,0001
DOID:1492	eye and adnexa disease	0,2815	< 0,0001	< 0,0001
DOID:688	embryonal cancer	0,3055	< 0,0001	< 0,0001
DOID:10591	pre-eclampsia	0,3647	< 0,0001	< 0,0001
DOID:8398	osteoarthritis	0,3879	< 0,0001	< 0,0001
DOID:1793	pancreatic cancer	0,3152	< 0,0001	< 0,0001

DOID:1883	hepatitis C	0,3522	< 0,0001	< 0,0001
DOID:0060100	musculoskeletal system cancer	0,2724	< 0,0001	< 0,0001
DOID:2355	anemia	0,3517	< 0,0001	< 0,0001
DOID:0060084	cell type benign neoplasm	0,2761	< 0,0001	< 0,0001
DOID:4766	embryoma	0,2988	< 0,0001	< 0,0001
DOID:10155	intestinal cancer	0,3050	< 0,0001	< 0,0001
DOID:1040	chronic lymphocytic leukemia	0,3692	< 0,0001	< 0,0001
DOID:3910	lung adenocarcinoma	0,3745	< 0,0001	< 0,0001
DOID:9256	colorectal cancer	0,3048	< 0,0001	< 0,0001
DOID:5672	large intestine cancer	0,3035	< 0,0001	< 0,0001
DOID:5082	liver cirrhosis	0,3558	< 0,0001	< 0,0001
DOID:6000	congestive heart failure	0,3341	< 0,0001	< 0,0001
DOID:201	connective tissue cancer	0,2709	< 0,0001	< 0,0001
DOID:9352	type 2 diabetes mellitus	0,3543	< 0,0001	< 0,0001
DOID:0080000	muscular disease	0,2657	< 0,0001	< 0,0001
DOID:3856	male reproductive organ cancer	0,2558	< 0,0001	< 0,0001
DOID:219	colon cancer	0,2944	< 0,0001	< 0,0001
DOID:423	myopathy	0,2683	< 0,0001	< 0,0001
DOID:66	muscle tissue disease	0,2683	< 0,0001	< 0,0001
DOID:1542	head and neck carcinoma	0,3110	< 0,0001	< 0,0001
DOID:10283	prostate cancer	0,2610	< 0,0001	< 0,0001
DOID:11934	head and neck cancer	0,3039	< 0,0001	< 0,0001
DOID:9970	obesity	0,2932	< 0,0001	< 0,0001
DOID:654	overnutrition	0,2886	< 0,0001	< 0,0001
DOID:5679	retinal disease	0,2789	< 0,0001	< 0,0001
DOID:9538	multiple myeloma	0,2975	< 0,0001	< 0,0001
DOID:10534	stomach cancer	0,3012	< 0,0001	< 0,0001
DOID:120	female reproductive organ cancer	0,2531	< 0,0001	< 0,0001

DOID:1612	breast cancer	0,2396	< 0,0001	< 0,0001
DOID:5093	thoracic cancer	0,2396	< 0,0001	< 0,0001
DOID:374	nutrition disease	0,2789	< 0,0001	< 0,0001
DOID:612	primary immunodeficiency disease	0,3364	< 0,0001	< 0,0001
DOID:2320	obstructive lung disease	0,2979	< 0,0001	< 0,0001
DOID:0060085	organ system benign neoplasm	0,2940	< 0,0001	< 0,0001
DOID:4905	pancreatic carcinoma	0,3033	< 0,0001	< 0,0001
DOID:870	neuropathy	0,3233	< 0,0001	< 0,0001
DOID:2043	hepatitis B	0,3240	< 0,0001	< 0,0001
DOID:2151	malignant ovarian surface	0,2777	< 0,0001	< 0,0001
DOID:2152	ovary epithelial cancer	0,2777	< 0,0001	< 0,0001
DOID:4001	ovarian carcinoma	0,2777	< 0,0001	< 0,0001
DOID:15	reproductive system disease	0,2736	< 0,0001	< 0,0001
DOID:657	adenoma	0,2674	< 0,0001	0,0001
DOID:5520	head and neck squamous cell carcinoma	0,3104	< 0,0001	0,0001
DOID:1036	chronic leukemia	0,3020	0,0001	0,0001
DOID:184	bone cancer	0,2694	0,0001	< 0,0001
DOID:10652	Alzheimer's disease	0,2302	0,0001	0,0002
DOID:3070	malignant glioma	0,2835	0,0001	0,0002
DOID:3083	chronic obstructive pulmonary disease	0,3078	0,0001	0,0002
DOID:8466	retinal degeneration	0,2740	0,0002	0,0002
DOID:680	tauopathy	0,2243	0,0002	0,0003
DOID:3459	breast carcinoma	0,2422	0,0002	0,0003
DOID:2394	ovarian cancer	0,2510	0,0007	0,0009
DOID:326	ischemia	0,2519	0,0034	0,0043
DOID:0050737	autosomal recessive disease	0,2300	0,0039	0,0049
DOID:3347	osteosarcoma	0,2464	0,0041	0,0051
DOID:1826	epilepsy syndrome	-0,3043	0,0073	0,0090
DOID:3093	nervous system cancer	0,1812	0,0125	0,0149

DOID:1575	rheumatic disease	0,2511	0,0126	0,0149
DOID:418	systemic scleroderma	0,2511	0,0126	0,0149
DOID:419	scleroderma	0,2511	0,0126	0,0149
DOID:854	collagen disease	0,2442	0,0145	0,0170
DOID:936	brain disease	0,1888	0,0297	0,0345
DOID:0050736	autosomal dominant disease	0,1882	0,0379	0,0437

Table S5.5. Enriched DO terms in genes co-expressed with DDAH2 in the dorsolateral prefrontal cortex of schizophrenic patients.

**ID**	**Description**	**Enrichment score**	***p*-value**	**p.adjust**
DOID:1115	sarcoma	0,3488	0,0001	0,0111
DOID:0050155	sensory system disease	0,2625	0,0004	0,0245
DOID:0050161	lower respiratory tract disease	0,2624	0,0016	0,0429
DOID:10763	hypertension	0,2494	0,0022	0,0429
DOID:850	lung disease	0,2640	0,0023	0,0429
DOID:5614	eye disease	0,2543	0,0025	0,0429
DOID:1579	respiratory system disease	0,2582	0,0027	0,0429
DOID:3393	coronary artery disease	0,2908	0,0027	0,0429
DOID:10591	pre-eclampsia	0,3001	0,0038	0,0470
DOID:5683	hereditary breast ovarian cancer	0,2947	0,0038	0,0470
DOID:18	urinary system disease	0,2484	0,0055	0,0476
DOID:557	kidney disease	0,2511	0,0056	0,0476
DOID:5844	myocardial infarction	0,3002	0,0057	0,0476
DOID:2348	arteriosclerotic cardiovascular disease	0,2671	0,0058	0,0476
DOID:1492	eye and adnexa disease	0,2514	0,0059	0,0476
DOID:2349	arteriosclerosis	0,2738	0,0062	0,0476
DOID:5679	retinal disease	0,2660	0,0065	0,0476
